# Vaccines and Therapies for Biodefence Agents

**DOI:** 10.1155/2015/537319

**Published:** 2015-03-08

**Authors:** Julia A. Tree, E. Diane Williamson, Caroline A. Rowland, Louise M. Pitt

**Affiliations:** ^1^Microbiological Services, Public Health England (PHE), Porton Down, Salisbury, Wiltshire SP4 OJG, UK; ^2^Biomedical Science Department, Defence Science and Technology Laboratory (DSTL), Porton Down, Salisbury, Wiltshire SP4 OJQ, UK; ^3^Virology Department, United States Army Medical Research Institute of Infectious Diseases (USAMRIID), Fort Detrick, Frederick, MD 21703, USA

This special issue, dealing with biodefence vaccines and therapies, incorporates two review papers and six original research papers. The review papers introduce concepts and themes underpinning the research papers, including the use of animal models to predict human responses in the transition of candidate vaccines and therapies from research to development and the impact of advances in genomic and imaging technologies on assessing the host immune response to biodefence agents.

Biothreat agents usually cause severe disease so clinical efficacy studies of candidate vaccines or therapies will generally not be ethical or feasible, unless in a field trial situation in a region of endemic disease. Thus there is a need to develop authentic models of the human disease in relevant* in vitro* and* ex vivo *cell culture and* in vivo* animal models and to apply these models to evaluate candidates. This process has been facilitated by the publication of the Animal Rule by the US Food and Drug Agency in 2002, through which a number of antibiotics have recently been approved for inhalational anthrax or plague indications, as have therapies for botulism and anthrax and a next generation vaccine for smallpox. More details about this can be found in the review by E. D. Williamson.

Biodefense agents are dangerous pathogens that pose unique challenges for researchers who often have to work at high biocontainment (levels 3 and 4) making routine experiments difficult to perform. By using various “state of the art” techniques such as optical imaging, biophotonic imaging, next generation sequencing, and microarrays, data derived from each experiment are greatly enhanced and the study of the host response to infection significantly improved. Recent progress using these “omic” and imaging technologies in biodefense research is reviewed by J. A. Tree et al.

Biodefense research benefits from improving and enhancing the utility of specific animal models. In this issue, M. Nelson and M. Loveday describe how they explored the innate immunological response of the common marmoset (*Callithrix jacchus*) to bacterial challenge. The immune response of this nonhuman primate is often understudied because of a lack of reagents. However, M. Nelson and M. Loveday examined the cross-reactivity of commercially available human reagents with marmoset markers and showed that it was possible to characterise the marmoset immune response to infectious disease in terms of phenotype, cell activation status, and key cytokine and chemokine expression. This is particularly useful because there is an increasing body of evidence that suggests that the physiological and immunological responses to biological agents are similar between marmosets and humans.

Progress in the approval of candidate vaccines and therapies is supported by ongoing research which improves the collective understanding of the pathogenesis of disease; two papers in this issue support this area. J. L. Dankmeyer et al. present results about the innate host response to* Yersinia pestis*. They suggest that Toll-like receptor 4 and the adaptor protein Myd88 are important for an optimal antibody response to the subunit F1-V plague vaccine and that Myd88 also appears to be required for protection against a lethal challenge in vaccinated mice. S. L. Newstead et al. describe the role of nitric oxide in the control of intracellular infection with* F. tularensis*. They infected two murine macrophage cell lines and demonstrated a differential ability to control the intracellular replication of the attenuated live vaccine strain of* F. tularensis* compared with the fully virulent SCHU S4 strain. Some control of SCHU S4 replication was achieved by preactivation of macrophages, indicating the involvement of factors additional to NO production in this effect.

In order for medical countermeasures to be licensed using the Animal Rule there is a need to bridge human immune responses to the animal models to identify surrogate markers of efficacy. This process is highlighted in the research paper modelling pneumonic plague, by V. A. Graham et al., in which an optimised murine model is described. Serum taken from human volunteers vaccinated with a new acellular plague vaccine (containing recombinant proteins rF1 and rV) was assessed for protective efficacy by passive transfer into mice. Mice were subsequently exposed to a lethal aerosol of plague and the delay in mean time-to-death measured. This process was shown to be reproducible and these data indicated that Hsd:NIHS mice may be a better model than BALB/c mice for passive transfer studies with human serum.

The last two papers in this special issue describe research on a generic DNA vaccination approach and a potential immunotherapy treatment for Ebola. S. Qiu and colleagues constructed DNA and recombinant Tiantan vaccinia (rTTV) vaccines encoding OVA-CTB fusion protein. They concluded that, following studies in mice, using an intranasal DNA prime and intramuscular recombinant-vaccinia boost, that fusion-expressed CTB could serve as a potent adjuvant to enhance both systemic and mucosal T-cell responses. S. Dowall et al. describe early studies using* in vitro* models to investigate potential targets for immunomodulatory therapy during infection with the highly infectious Ebola virus. They showed binding of the phosphatidylserine-targeting antibody (bavituximab), which has already progressed through clinical trials (phases I-III) for treatment of tumours, to the surface of host cells infected with Ebola and also to purified Ebola virions. Assessing immune therapies for other indications has the potential to allow repurposing of products for treatment of biodefence agents, potentially saving significantly on research and development time and costs.

In conclusion, this special issue has surveyed a diverse range of approaches to the development of biodefence vaccines and therapies and has explored the likely impact and application to the field of exciting new technological developments.

*Julia A. Tree* *Julia A. Tree* *E. Diane Williamson* *E. Diane Williamson* *Caroline A. Rowland* *Caroline A. Rowland* *Louise M. Pitt* *Louise M. Pitt*

